# Editorial: Quantitative analysis of cranial ultrasound for brain injury in premature infants

**DOI:** 10.3389/fneur.2023.1308333

**Published:** 2023-10-23

**Authors:** Xiaodong Yang, Akram Alomainy, Qammer H. Abbasi

**Affiliations:** ^1^School of Electronic Engineering, Xidian University, Xi'an, Shaanxi, China; ^2^School of Electronic Engineering and Computer Science, Queen Mary University of London, London, United Kingdom; ^3^James Watt School of Engineering, University of Glasgow, Glasgow, United Kingdom

**Keywords:** quantitative analysis, premature infant, cranial, medical image, comparison

The brain injury and various developmental abnormalities of premature infants are usually not very obvious and are often overlooked due to the lack of obvious symptoms, the related complications seriously affect the quality of life of premature infants. Early diagnosis, intervention, and efforts to promote the intellectual development of premature infants are important means to reduce disease and improve prognosis. Medical imaging analysis plays an important role in the fields of premature brain injury analysis, premature risk factors analysis, premature brain volume analysis, and premature neural development analysis. Ultrasound imaging, MRI and CT imaging have their own advantages and complement each other in the analysis of craniocerebral injury in premature infants. In the analysis of craniocerebral injury in premature infants, comprehensive consideration should be given according to the specific situation.

The first study, Zhang and Zhou(a), focused on the processing of brain ultrasound images of premature infants. The algorithm framework in this paper is mainly composed of image preprocessing, region of interest extraction and image quantitative analysis. According to the characteristics of the ultrasound image itself (including noise and artifacts), the authors adopted the method of adaptive median filtering. On the one hand, the noise is effectively suppressed, on the other hand, the micro tissue information is preserved. This algorithm can match the corresponding filtering window according to the change of noise, and its adaptive characteristics have obvious advantages. On the basis of expounding the basic idea of the algorithm, the paper also describes the principle and implementation process of the algorithm, and gives the brain ultrasound image of premature infants after adaptive median filtering. It can be seen from the image that the expected effect has been achieved. The ultrasound image after adaptive median filtering needs to further enhance the contrast to highlight the details in the image. In this paper, the authors used the CLAHE method. In this paper, the basic idea, implementation steps and basic principles of the method are described, and the effect diagram processed by the method is given. The extraction of the region of interest is a necessary step in the analysis of brain ultrasound images of premature infants. This paper is mainly realized by manual interaction. The final step is the quantitative analysis of brain ultrasound images of premature infants. The algorithm involves the concepts of gray mean and gray level co-occurrence matrix. The paper briefly describes the basic steps of quantitative analysis and the mathematical implementation method, and calculates the relevant feature quantities. Finally, the quantitative analysis results and related tables under different planes are given, and the brain ultrasound image analysis of the subjects is completed. This study has certain reference significance for the analysis of craniocerebral ultrasound images of premature infants.

The second study, Liu, summarized and discussed the analysis of brain injury in premature infants through medical ultrasound images. This paper introduces the imaging principle of craniocerebral ultrasound in premature infants and its unique advantages. At the same time, the paper also discusses the related preparation work that needs to be carried out before brain ultrasound examination in premature infants, and the classification according to different imaging sites. Different imaging sites target different brain structures and tissues. On this basis, the paper also discusses the timing of the inspection, and gives the recommended inspection time and interval. Finally, the paper classifies the ultrasonic diagnosis of different types of brain injury in premature infants, and discusses several typical brain injuries in premature infants, including hypoxic-ischemic encephalopathy, periventricular leukomalacia, and intracranial hemorrhage. Through a comprehensive discussion, this paper makes readers have a further understanding of the knowledge in related fields.

The third study, Zhang and Zhou(b), comprehensively discussed the characteristics of several medical imaging and their application in the analysis of brain injury in premature infants. Starting from the definition and etiology of brain injury in premature infants, this paper gradually introduces several representative medical imaging methods, including ultrasound, MRI and CT. On this basis, the diagnostic value of different imaging methods in brain injury of premature infants was described. For ultrasound imaging, the resolution, non-invasive, bedside detection, real-time, safety and other aspects are discussed, and the typical symptoms of brain injury in premature infants in different periods are given. For CT imaging, its role and precautions in brain injury of premature infants are given. In abnormal cases, it can be used as an effective supplement to ultrasound imaging methods. For MRI, the paper first introduces a variety of different types of improved imaging methods, and discusses the application of this imaging technique in the analysis of brain injury in premature infants in detail in combination with different types, different locations, and different periods of brain injury in premature infants. Finally, the paper summarizes the characteristics and applicable scenarios of several imaging methods.

The fourth study, Kvanta et al., gives a new perspective. The paper adopts the method of voxel-based morphometry, and is a continuation of the previous research of the group. The high-quality magnetic resonance images are considered, and the regional brain volume alterations patterns of the relevant subjects are analyzed. In this paper, the relevant statistical analysis methods are discussed in detail, and the in-depth and effective analysis is carried out in combination with the image pictures. Through rigorous comparison and analysis, the important conclusions about the alterations of regional brain volume were obtained, which laid an important foundation for the related research and had important reference significance.

The neurological sequelae caused by brain injury in premature infants seriously affect the quality of life of premature infants, which brings a heavy burden to families and society. This Research Topic collects relevant manuscripts for such problems. These works comprehensively described the application of different medical imaging methods in the analysis of brain injury in preterm infants from a more comprehensive perspective, and gave the scope of application and unique advantages of different medical imaging methods. On the basis of qualitative analysis, further quantitative analysis was carried out to predict the neurodevelopmental outcome of premature infants at an earlier stage. The papers collected in the Research Topic and the ideas and methods involved have important reference significance for the diagnosis of neurodevelopmental disorders in premature infants and early intervention of brain injury in premature infants, which can alleviate the resulting emotional anxiety and reduce the cost of repeated visits.

## Author contributions

XY: Writing — original draft. AA: Writing — review & editing. QA: Writing — review & editing.

